# Comparison of Two Rift Valley Fever Serological Tests in Cameroonian Cattle Populations Using a Bayesian Latent Class Approach

**DOI:** 10.3389/fvets.2019.00258

**Published:** 2019-08-14

**Authors:** Barend M. C. de Bronsvoort, Jean-Marc Bagninbom, Lucy Ndip, Robert F. Kelly, Ian Handel, Vincent N. Tanya, Kenton L. Morgan, Victor Ngu Ngwa, Stella Mazeri, Charles Nfon

**Affiliations:** ^1^The EERA Group, The Roslin Institute and Royal (Dick) School of Veterinary Studies, University of Edinburgh, Easter Bush, Midlothian, United Kingdom; ^2^The Royal (Dick) School of Veterinary Studies, University of Edinburgh, Easter Bush, Midlothian, United Kingdom; ^3^School of Veterinary Medicine and Sciences, University of Ngaoundere, Ngaoundere, Cameroon; ^4^Laboratory of Emerging Infectious Diseases, University of Buea, Buea, Cameroon; ^5^Programme Office, Cameroon Academy of Sciences, Yaoundé, Cameroon; ^6^Regional Centre of Wakwa, The Institute of Agricultural Research for Development, Ngaoundere, Cameroon; ^7^The Institute of Aging and Chronic Disease, The School of Veterinary Science, University of Liverpool, Neston, United Kingdom; ^8^National Centre for Foreign Animal Disease, Winnipeg, MB, Canada

**Keywords:** Cameroon, no gold standard, sensitivity, specificity, Rift Valley fever (RVF)

## Abstract

Rift Valley Fever is an important zoonotic viral disease of livestock occurring across much of Africa causing acute febrile illness, abortion, and neonatal death in livestock particularly sheep and cattle and a range of disease in humans from mild flu-like symptoms to more severe haemorrhagic fever and death. Understanding the epidemiology requires well-evaluated tools including antibody detection ELISAs. It is well-recognized that tests developed in one population do not necessarily perform as well when used in different populations and it is therefore important to assess tests in the populations in which they are to be used. Here we describe the performance of a commercial RVF ELISA (ID.Vet) and an in-house plaque reduction neutralization test (PRNT_80_). A Bayesian no gold standard latent class model for two tests and ≥2 populations based on the Hui-Walter model was used to estimate the test parameters using a range of populations based on geographical separation and age to assess consistency of performance across different sub-populations. The ID.Vet ELISA had an estimated diagnostic sensitivity (Se) of 0.854 (0.655–0.991 95%BCI) and specificity (Sp) of 0.986 (0.971–0.998 95%BCI) using all the data and splitting the population by geographical region compared to 0.844 (0.660–0.973 95%BCI) and 0.981 (0.965–0.996 95%BCI) for the PRNT_80_. There was slight variation in the mean Se and Sp in different sub-populations mainly in Se estimates due to small numbers of positives in the sub-populations but the 95% BCI generally overlapped suggesting a very consistent performance across the different geographical areas and ages of animals. This is one of few reports of serological evidence of RVF in Central Africa and strongly suggests the virus is actively circulating in this cattle population. This has important public health implications and RVF should be considered as a differential in both livestock disease cases as well as human febrile cases in West and Central Africa not just East Africa. We also demonstrate that the performance of the commercial ELISA is comparable to the PRNT_80_ but has the advantages of speed, lower cost and no containment needs making it a much more useful test for low and middle income settings (LMICs).

## Introduction

Rift Valley Fever (RVF) is a mosquito-borne zoonotic viral disease of ruminants, caused by a *Phlebovirus* in the *Bunyaviridae* family. It was first described in Kenya in 1931, and has since been reported in many African countries, as well as the Arabian Peninsula (1–3). It is considered one of the most important emerging zoonotic pathogens of public health significance affecting mainly African communities with low resilience to economic and environmental challenges (4). The epidemiology is characterized by explosive epidemics in both humans and livestock populations usually associated with flooding or dam construction and long inter-epidemic periods where there is little evidence of viral presence in those populations affected by epidemics. Where the virus persists in these inter-epidemic periods is still a major gap in our understanding of the epidemiology of RVF (4).

*Aedes* and *Culex* mosquitoes are the main vectors of the RVF virus (RVFV), and it can be transferred vertically from female mosquitoes to their eggs in some species of the *Aedes* genera (5–7). Sheep, goats, and cattle are the domestic species most affected but clinical signs are usually mild and inapparent in adult animals but can lead to major outbreaks of abortions and death in neonates during epidemic periods which result in direct significant economic losses (5, 8, 9). The disease can also affect other wild animals such as buffalo, as well as spill over into humans (10). RVF is transmitted between animals and to humans through the bite of an infected mosquito vector. The disease in humans can also result from direct contact with infected tissues, blood or body fluids (11). A rise in RVFV prevalence in domestic ruminants can sometimes precede epidemics in humans (1) and similarly a decline in herd immunity in the inter-epidemic periods coupled with extensive flooding appears to facilitate these explosive outbreaks. Symptoms of the disease in humans can vary, ranging from flu-like symptoms to more severe conditions such as meningoencephalitis, haemorrhagic fever, or death (5, 11). The case fatality rate for patients developing the haemorrhagic form of the disease can be as high as 50% (4).

Epidemiological studies have focused upon East Africa (12) where the virus was first isolated, with less known about its significance in Central-West African human or livestock populations although outbreaks in human populations in West Africa have been associated with dam projects (4). Within the Central African region, livestock and human cases of RVF have been reported in the savanna of northern Cameroon, Chad, and within forest areas in the Central African Republic. Livestock seroprevalences of 9–20% within goat herds of northern Cameroon (1, 13) and 4.4% in cattle, 10.7% in sheep and 8.6% in goats in Chad (14) have been reported. Most recently, in a large sample across Cameroon prevalence estimates of 13.5% (11.4–15.7) for cattle and 3.4% (2.3–4.7) for small ruminants were produced (15).

Cameroon is a significant cattle producer of the Central-African region with livestock contributing ~$476 million to the national economy in 2010 (16) and being of cultural importance to rural communities. The Northwest Region (NWR) and the Vina Division (VD) of the Adamawa Region of Cameroon are major cattle keeping areas in the wider Adamawa Plateau of Central Africa. Cattle are kept for many reasons, including financial, draft power, dairy products, and trade. The area is mostly covered by sparse tree savannah, with a dry season between November and April, and the wet season from May until October (17). *Culex spp*. and *Aedes* spp. mosquitos are present in Cameroon and due to the close association between cattle and people in Cameroon, cattle may act as a reservoir for RVF although little is known about its epidemiology in this setting (15).

A number of tests have been developed to detect IgM and IgG antibodies in different species including a new commercial multi-species ELISA from ID.Vet (Montpellier, France) (15) and an in-house plaque reduction neutralization test by the Canadian (18). There are currently no validation studies in African cattle populations. It is well-recognized that diagnostic tests developed and validated in one population behave differently when used in different settings (19, 20). This is important for surveillance activities or risk factor evaluation in order for estimates at the population level to be correctly adjusted for the test imperfections and reliable estimates generated for evidence based decision making. Using population based serum banks from studies in Cameroon we are able to estimate test performance in naturally infected populations with a range of coinfections that may impact performance and thus get more reliable point estimates as well as capturing the variation and thus the uncertainty of these estimates.

Hui and Walter (21) developed a no gold standard model to estimate test sensitivity and specificity in the absence of a gold standard test under certain assumptions. These assumptions are that each test performs the same in each population, that the tests are conditionally independent (i.e., if a sample is positive in one test this does not influence the probability it will be positive on the other test conditional on its true status) and that the prevalences are different in each of the sampled populations (or the problem can become mathematically non-identifiable). Applying the model in a Bayesian framework and making some adjustment for the possible conditional dependence between tests (22) allows us to estimate the test parameters (sensitivity and specificity) as well as the population prevalence.

The aims of this study were to estimate the test performance of two RVF diagnostic tests in a naturally infected population and to assess the stability of these estimates in different geographical regions and age groups. In particular we were interested in understanding how the ID.Vet test performed as it has wide potential use as a simpler screening serological test for low and middle income settings (LMICs). Secondly to describe the seroprevalence of RVF exposures in cattle in the NWR and VD of Cameroon in 2013.

## Materials and Methods

In reporting this analysis the authors have followed the STARD (23) and recent STARD-BLCM (24) guidelines for reporting diagnostic test accuracy.

### Study Sites

The study was conducted in two sites in the NWR and VD of the Adamawa Region of Cameroon. Both are of similar geographical size of ~17,000 km^2^. The NWR is situated in the fertile mountainous highlands, 500–3,000 m above sea level. Bamenda, the capital, is Cameroon's third largest city. The Region is densely populated (1,804,695 people) and an estimated 506,548 cattle are grazed there (25). The VD is part of the fertile Adamawa Region's savannah plateau. The regional capital is Ngaoundere and the population of the VD (317,888 people) is much smaller than that of the NWR. The cattle population is also smaller with an estimated 176,257 head (26). Veterinary services are predominately provided by the government through the Ministry of Livestock, Fisheries, and Industrial Agriculture/Ministere de l'Elevage des Peches et Industries Animales (MINEPIA), with local veterinary technicians stationed at Zootechnical and Veterinary Sanitary Control Centers (ZVSCC) distributed across the country (17). Their responsibilities include registration of local livestock keepers, disease control mainly through annual vaccination campaigns, meat inspection, and regulation of livestock markets and animal movements.

### Study Design

A cross sectional survey was conducted between January–May 2013 in the NWR and September–November 2013 in the VD. These were pastoralists whose herds were listed in the Ministry of Livestock, Fisheries, and Animal Industries vaccination records at 81 local veterinary centers in the NWR and 31 in the VD in 2012. A total of 5,053 pastoralist herds in the NWR and 1,927 in the VD, with a range of 1–215 cattle per herd were included in the sampling frame. The list of herds in each site was stratified by administrative area; seven Divisions in the NWR and eight sub-Divisions within the VD and a random sample of herds was taken from each site proportional to the total number of herds listed in each of the two sites. This survey was part of a larger study of bovine tuberculosis and liver fluke and the sample size was based on a clustered random sample of cattle assuming a cattle level prevalence of ~10%, a within herd variance of 0.15 and between herd variance of 0.01, an average herd size of 70, a relative cost of 12:1 for herd:cattle and relative error of ±15% (Survey Toolbox; AusVet) (27). This gave a target sample size of 15 cattle per herd and 88 herds under the simplifying assumption of perfect test performance. To allow for potential losses or drop out and to have balanced samples from the two sites, we aimed for 50 herds in each of the two sites in the NWR and VD. Within each herd the 15 samples were stratified to each of three age classes; <2 years old (young), 2–5 years old (adult), and older than 5 years (old).

### ID Screen^®^ Rift Valley Fever Competition Multi-Species ELISA

The competitive ELISA was performed according to the instructions of the manufacturer and all the samples were run once. In brief, 50 μl of sample diluted 1:1 with the supplied kit buffer was added to each test well of the recombinant RVF nucleoprotein precoated plate and incubated for 1 h at 37°C. The plate was washed and 100 μl of the supplied anti-RVF-NP conjugate added and incubated for a further 30 min at 21°C and washed. 100 μl of supplied substrate solution supplied was added and incubated for a final 15 min at 21°C before adding the stop solution. The plate was read at 450 nm. To control the validity of each plate, the mean value of the two negative controls (ODNC) was calculated and the plate was considered valid when OD_NC_ > 0.7. For a valid plate, the mean value of the two positive controls divided by OD_NC_ should be <0.3. For each sample the competition percentage was calculated by dividing (OD_sample_/OD_NC_) × 100. The manufacturers suggest if the value was ≤ 40% the sample was considered positive. A value >50% was a considered a negative result and values in between 40 and 50% indicated an inconclusive result.

### RVF PRNT_80_

RVFV strain ZH501 (28) was propagated in a mosquito cell line (C6/36, ATCC) as previously described (18). Briefly, C6/36 cells were infected at a multiplicity of infection (MOI) of 0.1 and maintained at 28°C in a 1:1 mixture of EMEM (Wisent) and ESF-921 (Expression Systems, Woodland, CA, USA) supplemented with 2.5% FBS, 25 mM HEPES and 1 mM sodium pyruvate. Vero E6 cells were used to determine the titers of RVFV as previously described (18).

Neutralizing antibody response to RVFV was determined by plaque reduction neutralization test (PRNT_80_) modified from a previously described protocol (5). Serial 2-fold dilutions of serum in DMEM were made starting from 1 in 40 to obtain duplicates of 100 μl/well for each serum sample. One hundred microliter of DMEM containing 100 PFU of RVFV was added to each serum dilution, mixed, and incubated at 37°C, 5% CO_2_, and 95% relative humidity for 1 h. Two hundred microliter of the virus/serum mixture was then transferred onto a 48-well plate containing confluent Vero E6 cell monolayer and incubated for another 1 h. An overlay of 1.75% carboxymethylcellulose in DMEM containing 0.3% BSA was then added to all wells and plates incubated at 37°C, 5% CO_2_, and 95% relative humidity. Assay of negative and positive control sera as well as a back titration of the virus was performed at the same time as the test sera. After 4–5 days the cells were fixed with 10% formalin, stained with 0.5% crystal violet and plaques counted. The reciprocal of the highest serum dilution that prevented at least 80% CPE was taken as the PRNT_80_ titer for that sample.

### Data Analysis

Hui and Walter (21) introduced a latent class approach to the evaluation of diagnostic tests in the absence of a “gold-standard.” The Hui-Walter paradigm requires two (or more) tests evaluated in two (or more) populations. This model assumes that: (i) the prevalence of the disease is different within each population; (ii) the tests have the same properties across populations; (iii) and the tests must be conditionally independent given the disease status.

The Bayesian version of the Hui-Walter model (29) assumes that for the *i*th subpopulation the counts (**O**_**i**_) of the different combinations of test results, +/+, +/–, –/+, and –/– for the two tests, follow a multinomial distribution:

$$\begin{array}{l} O_{\text{i}}|{\text{Se}}_{\text{j}},{\text{Sp}}_{\text{j}},{\text{p}}_{\text{i}}\sim \text{Multinominal}(Pr_{\text{i}},{\text{n}}_{\text{i}})\text{ for i}=1,2,\ldots ,\text{S and}\\ \text{ j}=1,2,\ldots ,\text{T} \end{array}$$

where S is the number of subpopulations, T is the number of tests and **Pr**_**i**_ is a vector of probabilities of observing the individual combinations of test results. Conditioning on the (latent) disease status, these probabilities can be specified using the sensitivity (Se) and specificity (Sp) of the tests and the prevalence (p) in subpopulations. As an example, for two tests the probability of observing both tests positive in the *i*th subpopulation is given as:

$$\text{Pr}\left({\text{T}}_{\text{1}}\text{+},{\text{T}}_{\text{2}}\text{+}\right)={\text{Se}}_{1}{\text{Se}}_{2}{\text{p}}_{\text{i}}+\left(1-{\text{Sp}}_{1}\right)\left(1-{\text{Sp}}_{2}\right)\left(1-{\text{p}}_{\text{i}}\right)$$

The other three probabilities for the remaining three test scenarios may be similarly derived.

In a Bayesian analysis all parameters are given distributions. Hence, prior distributions for the test properties and the prevalences within the subpopulations must be specified. For prevalences where no information was available the distributions were modeled using a uniform distribution on the interval between 0 and 1 with a beta(1,1) distribution. The Se and Sp of the two tests were modeled using the priors Se_1_~Beta(20,1) and Sp_1_~Beta(5,1) for the ID.Vet ELISA based on published estimates of performance (30) and vaguer priors Se_2_~Beta(5,2) and Sp_2_~Beta(10,2) were used for the RVF PRNT_80_. A sensitivity analysis was done to confirm that the priors were not overwhelming the posteriors and driving the estimates (see Supplementary Material S3).

If the two tests cannot be reasonably assumed to be independent then the Hui-Walter model must be extended (19, 31) to account for the covariance structure between the two tests as below:

$$\begin{array}{r} \text{Pr}\left({\text{T}}_{\text{1}}\text{+},{\text{T}}_{\text{2}}\text{+}\right)={\left(\left({\text{Se}}_{\text{1}}^{*}{\text{Se}}_{\text{2}}\right)\text{+ covDp}\right)}^{*}{\text{p}}_{\text{i}}\text{+ }{\left(\left({\left(\text{1}-{\text{Sp}}_{\text{1}}\right)}^{*}\left(\text{1}-{\text{Sp}}_{\text{2}}\right)\right)\text{+ covDn }\right)}^{*}\left(\text{1}-{\text{p}}_{\text{i}}\right)\\ \text{Pr}({\text{T}}_{\text{1}}\text{+},{\text{T}}_{\text{2}}-)={\left(\left({\left({\text{Se}}_{\text{1}}\right)}^{*}\left({1-\text{Se}}_{\text{2}}\right)\right)\;-\text{covDp}\right)}^{*}{\text{p}}_{\text{i}}\text{+}\;{\left(\left({\left(\text{1}-{\text{Sp}}_{\text{1}}\right)}^{*}{\text{Sp}}_{\text{2}}\right)\;-\text{covDn}\right)}^{*}\left(\text{1}-{\text{p}}_{\text{i}}\right)\\ \text{ Pr}({\text{T}}_{\text{1}}-,{\text{T}}_{\text{2}}\text{+})=\left({\left(\text{1}-{\text{Se}}_{\text{1}}\right)}^{*}{\text{Se}}_{\text{2}}\right){-\text{ covDp})}^{*}{\text{p}}_{\text{i}}\text{+ }{\left(\left({\text{Sp}}_{\text{1}}{\;}^{*}\left(\text{1}-{\text{Sp}}_{\text{2}}\right)\right)-\text{covDn}\right)}^{*}\left(\text{1}-{\text{p}}_{\text{i}}\right)\\ \text{ Pr}\left({\text{T}}_{\text{1}}-,{\text{T}}_{\text{2}}-\right)={\left({\left(\text{1}-{\text{Se}}_{\text{1}}\right)}^{*}\left(\text{1}-{\text{Se}}_{\text{2}}\right)\text{+ covDp}\right)}^{*}{\text{p}}_{\text{i}}\text{+ }{\left(\left({\text{Sp}}_{\text{1}}^{*}{\text{Sp}}_{\text{2}}\right)\text{+ covDn}\right)}^{*}\left(\text{1}-{\text{p}}_{\text{i}}\right) \end{array}$$

The covDp and the covDn are the covariances between the two tests when the animal is diseased and when it is not diseased, respectively. The covariance between the test outcomes for infected subpopulations satisfies (Se_1_-1)^*^(1-Se_2_) ≤ covDp ≤ (min(Se_1_,Se_2_)-(Se_1_Se_2_)) and for the non-infected subpopulation, (Sp_1_-1)^*^(1-Sp_2_) ≤ covDp ≤ (min(Sp_1_,Sp_2_)-(Sp_1_Sp_2_). Therefore, for instance, a uniform ((Se_1_-1)(1-Se_2_), (min(Se_1_,Se_2_)-(Se_1_Se_2_))) prior distribution can be used for covDp.

The model was implemented in JAGS using R (32) (code available in Supplementary Material S1). For this analysis the chains were used and the first 50,000 iterations were discarded as a burn-in. A further 250,000 iterations were run for each chain and then thinned by 100 to produce a set of 7,500 interactions kept for posterior inference. The parameter estimates are the mean of the posterior and the 0.025 and 0.975 quantiles were used to give the Bayesian credibility intervals (BCI). Convergence of the chain after the initial burn-in was assessed by visual inspection of the time-series plots for the parameters, as well as Gelman-Rubin statistic and diagnostic plots of model convergence based on the three sample chains with dispersed starting values (33).

### Descriptive Statistics and Mapping

All statistical modeling and data visualization was conducted in R 3.5.2 (34).

## Results

**Figure 1:**
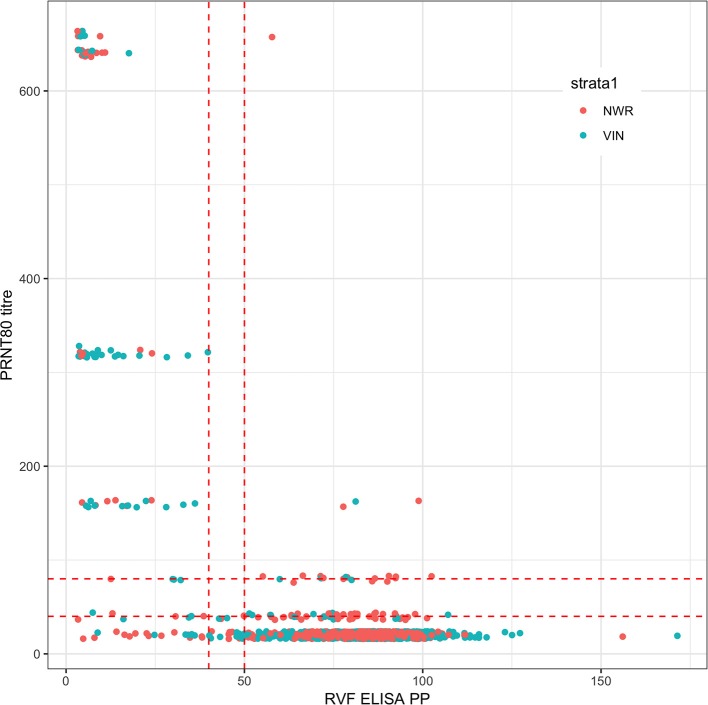
Bivariate plot of the raw continuous values from the PRNT_80_ and ID.Vet RVF tests. The points are colored by study site and jittered on the y axis. Horizontal and vertical lines in dashed red were added to mark the various cut-off values used. Note that for the PRNT_80_ a positive result is greater than the cut-off while for the ID.Vet ELISA is lower than the cut-off.

A total of 1,498 cattle were sampled across the 2 study sites (January–May 2013 in the NWR and September-November 2013 in the VD). Eighteen samples failed the PRNT_80_ test and a further seven failed the ID.Vet test due to excessive hemolysis leaving a final sample size of 1,473 for the remaining analyses. The raw continuous readings from the two tests are presented in Figure 1 and there is generally good agreement.

**Figure 2:**
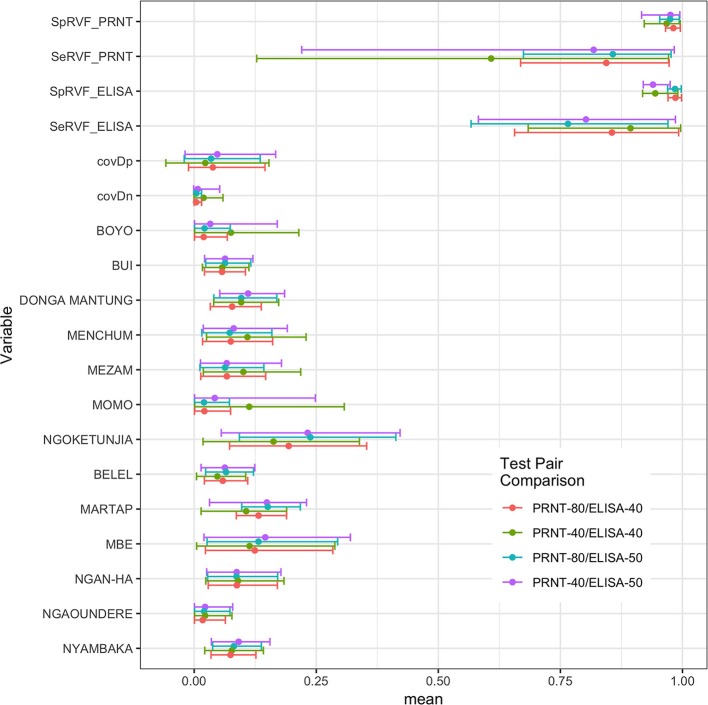
Bayesian posterior means and 95% BCI for the two tests and their covariances (covDn, covDp) and the 13 prevalences for the subpopulations using the Hui-Walter NGS model allowing for conditional dependence between tests. The four test/cut-off combinations are presented together for comparison.

An initial NGS analysis was conducted comparing the four different combinations of tests/cut-offs to identify the best combination for the rest of the analysis. The data was subset into 13 different populations based on the administrative Divisions (NWR) or sub-Divisions (VD) used in the sampling design. The posterior estimates for the test parameters and the different population prevalences are shown in Figure 2. From this the specificities are all very high with narrow 95% BCIs but as might be expected using the lower cut-off for the PRNT_80_ in particular results in lowered specificities. The sensitivities are all lower than the specificities and have a much higher uncertainty around the estimates reflecting the relatively small number of positives in the sample (for all cut-offs used). Interestingly, all sets of four prevalence estimates for each population are very consistent across the test combinations. Overall the combination with the ELISA cut-off set at 40pp or lower and the PRNT_80_ at 80 or greater were considered the optimal cut-offs as they produced on average the highest sensitivity and specificity estimates for both tests (Table 1).

**Table 1:** No gold standard estimates of the sensitivity (Se) and specificity (Sp) and Bayesian 95% credibility intervals (BCI) for the ID.Vet Rift Valley Fever ELISA at a cut-off of 40 and the in house PRNT_80_ with a cut-off of 80.

**Parameter**	**Mean**	**95% BCI**
Se IDVet ELISA	0.854	0.655–0.991
Sp IDVet ELISA	0.986	0.971–0.998
Se PRNT_80_	0.844	0.660–0.973
Sp PRNT_80_	0.981	0.965–0.996

**Figure 3:**
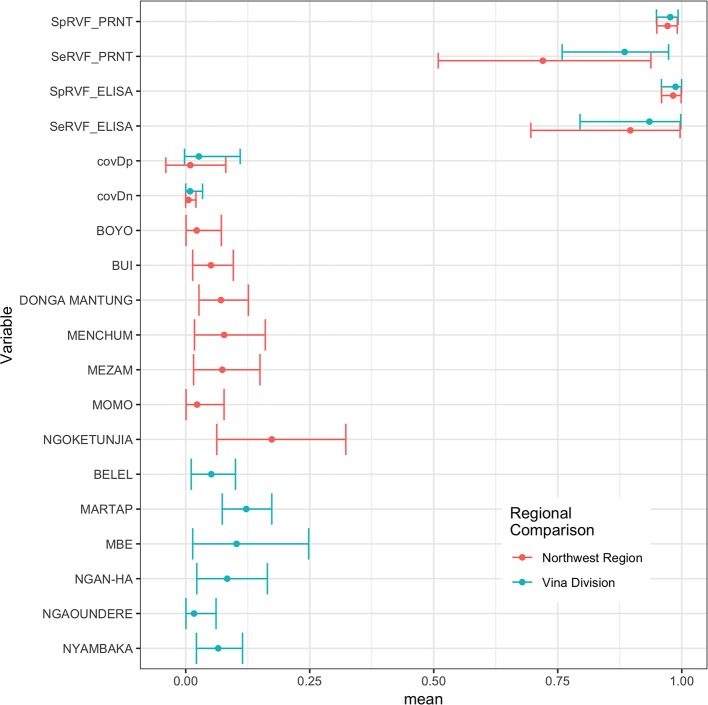
Bayesian mean and 95% BCI for the ID.Vet ELISA (cut-off 40pp) and the PRNT_80_ (cut-off 80) estimated independently in the two study sites in the Northwest Region and Vina Division.

**Figure 4:**
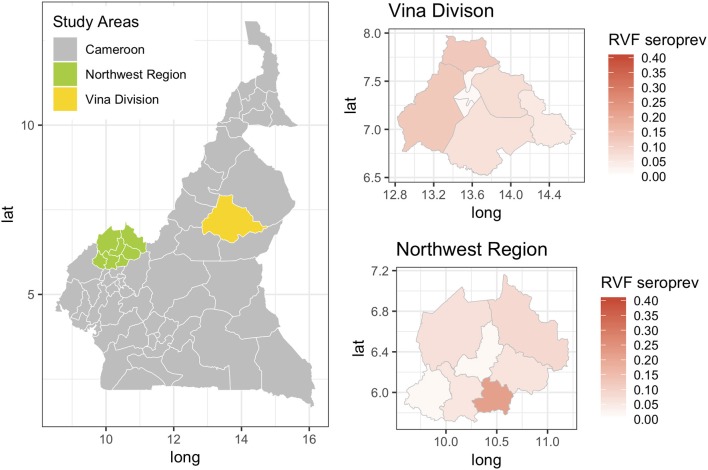
Map showing the two study sites in the Northwest Region and the Vina Division of Cameroon. The smaller chloropeth maps show the mean estimated prevalence of rift valley fever seroprevalence for each site by Division (NWR) or sub-Division (VD).

The Hui-Walter model assumes that the test performs the same in each population. To explore this we repeated the analysis at a site level running independent models for the NWR and VD. The resulting estimates are given in Figure 3 and show that although these is some variation between the sites the specificities remain very high and the sensitivities a little lower with considerable uncertainty as might be expected from the smaller sample sizes but overall a very similar estimate across the two study sites. The prevalence estimates are almost identical to those from the single model estates shown in Figure 1. These have also been plotted on a map in Figure 4 to highlight the spatial variation in seroprevalence across the two sites.

**Figure 5:**
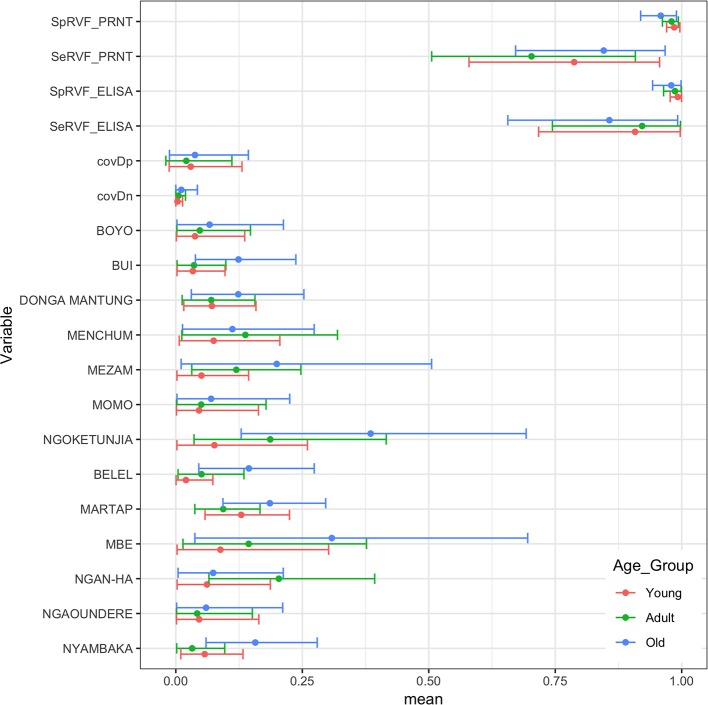
Bayesian mean and 95% BCI parameter estimates for the ID.Vet RVF ELISA (cut-off 40pp) and the RVF PRNT80 (cut-off 80) estimated independently in the three age classes of cattle in Cameroon and the age class specific seroprevalence estimates across the 13 subpopulations.

**Figure 6:**
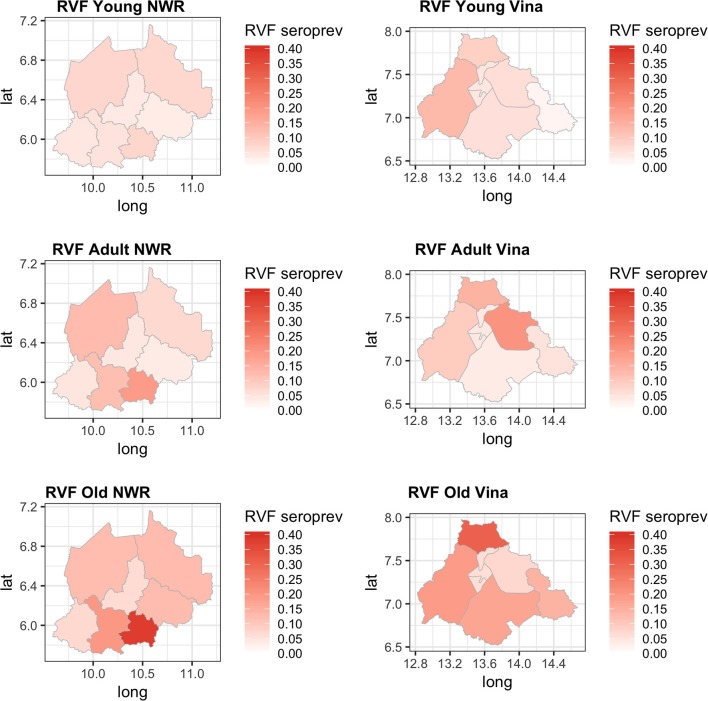
Chloropeth maps showing the two study sites of the Northwest (Divisions) and Vina Division (sub-Divisions) of Cameroon and the Bayesian NGS mean seroprevalence estimates for the three age classes from young adult and old.

Finally, we were interested to compare the performance of the tests in different age groups. The cattle were classified as young (<2 years old), adult (≥2 but <5 years old), and old (≥5 years old) based on the age and or dentition collected at the time of the sampling. The estimates based on the two tests and 13 sub-populations run as a single model are given in Figure 5 with the test parameters further highlighted in Table 2. Again both tests appear to perform very consistently across the different age groups although the specificity seems to drop slightly in the old class for both tests for reasons that are not clear but which are reflected in higher levels of misclassification/lower agreement in this age class. It is also reassuring that seroprevalence generally increases with age group as one would expect for a vector borne infectious disease (Figures 5 and 6).

**Table 2:** No gold standard estimates of the sensitivity (Se) and specificity (Sp) with Bayesian 95% credibility intervals (BCI) for the ID.Vet Rift Valley Fever ELISA at a cut-off of 40 and the in house PRNT80 with a cut-off of 80 estimated separately for each age group.

**Parameter**	**Age class**	**Mean**	**95% BCI**
Se ID.Vet ELISA	Young	0.906	0.712–0.997
	Adult	0.941	0.817–0.998
	Old	0.857	0.655–0.992
Sp ID.Vet ELISA	Young	0.992	0.977–1.000
	Adult	0.990	0.973–1.000
	Old	0.979	0.944–0.999
Se PRNT_80_	Young	0.786	0.577–0.956
	Adult	0.754	0.567–0.937
	Old	0.847	0.672–0.968
Sp PRNT_80_	Young	0.985	0.970–0.996
	Adult	0.985	0.971–0.995
	Old	0.958	0.920–0.989

## Discussion

Rift Valley fever is an important emerging zoonotic disease in sub-Saharan Africa whose epidemiology is still poorly understood and there is a need to evaluate low cost surveillance tools for LMIC settings such as Cameroon. Here we have compared 2 RVF diagnostic tests, an in-house PRNT and a commercial ELISA (ID.Vet) using a latent class approach in a Bayesian framework to deal with the problem of having no gold standard test. The analysis first compared the tests using two different cut-offs and identified the optimal combination of cut-offs for the two tests that were then used for the remainder of the analyses (Supplementary Table S2). We also then compared their performances in geographically separated populations and also across age groups to assess consistency of performance and parameter estimates across these different groups. The results suggest that the tests perform consistently across the different groups with precise estimates of very high specificities and good sensitivities but with more uncertainty in these. There is some variation across the different subpopulation analyses, particularly in sensitivity estimates. This may be due to the relatively small numbers of positive animals per administrative area which results in stochastic noise when the sample is sub-setted by site and age.

The model assumes that the tests perform the same in the different populations and the results support this when the sample was split and analyzed separately for each site (Figure 3). We have included conditional dependence since both tests are serology based as suggested by Toft et al. (29). In addition, Toft et al. (29) warn about the potential inflation of the standard errors when the difference between the prevalences in the different subpopulations are small. In this study the range of prevalences is fairly wide therefore this should not be an issue. Finally, the model assumes all individuals are independent and does not account for clustering by herd which may lead to underestimation of the standard error and narrower BCIs. The specificity estimates are similar to those previously published for both the ID.Vet RVF ELISA (0.997) (30) and PRNT_80_ (0.960) (35). The estimates for the sensitivity of the ID.Vet RVF ELISA are lower than those published by Paweska et al. (30) who report a sensitivity of 0.963. Our results suggest that in this population it may perform less well but may be a better reflection of performance in a naturally infected population where we do not know the stage of infection and their is a wider range of infectious doses. Other ELISA tests are available including another competitive ELISA produced by Veterinary Medicine Research and Development (Washington State) which when compared to the PRNT_80_ had similar sensitivities and specificities (36).

This represents the first report of estimating the ID.Vet RVF ELISA performance in an African cattle population and the ELISA shows great potential as a low cost easy to use surveillance tool with very good overall accuracy comparable to the more standardized PRNT_80_. Although generally considered the reference test and widely used in reference laboratories the PRNT_80_ is labor-intensive, time consuming, expensive, and requires virus appropriate biocontainment (36) and so it not practical for most surveillance activities particularly in LMICs. Laboratories with only very basic equipment can use the kit making it very appropriate for use in Africa.

Previous studies have demonstrated that RVF virus is circulating in Cameroon and the Central African region more widely. Further, the results presented in this paper suggest that the seroprevalence of RVF in cattle varies across the study sites between 2 and 12% with the exception of Ngoketunjia in the NWR where it was particularly high at near 20%. This Division is to the south of the Region and includes a large dam and swamp areas where cattle are grazed and it would be consistent with much higher vector populations. There is also a clear pattern of increasing seroprevalence with age across the 13 sub-populations over the two sites consistent with viral circulation although no clinical disease has been reported. This may be because at the time of the study there was no animal surveillance for abortions. These estimates appear consistent with those recently reported from Cameroon for the Adamawa Region in 2018 (15) and 1995 (13). They do not report seroprevalences in cattle for the NWR. From discussions with local health personnel there is also very limited diagnostic exploration of human febrile illnesses which may lead into cases being missed. Given that there appears to be viral circulation, screening of high risk human groups such as slaughterhouse workers or veterinarians for evidence of exposure should be prioritized to provide further evidence for the need to include screening for RVF in febrile cases. Furthermore, RVF outbreaks have been associated with dam construction in West Africa and El Nino events in East Africa and changes in habitat that favor mosquito populations are a potential risk for triggering epidemics (37). Interestingly the highest seroprevalence in this study was from Ngoketunjia which includes a dam. With new dams currently under construction in Cameroon the potential risks for RVF outbreaks should be considered.

In conclusion we have demonstrated that both the ID.Vet ELISA and PRNT_80_ have comparable performances in cattle from Cameroon. This supports the use of the ELISA as a relatively low cost easy to use surveillance tool for the African context. The results also suggest that RVF virus is endemic and circulating in Cameroonian cattle and it is interesting that no clinical reports exist for cattle or humans. The results here and from other studies in Cameroon suggest that human screening of febriles illnesses should be considered by hospitals where malaria has been ruled out.

## Data Availability

The datasets generated for this study are available on request to the corresponding author.

## Ethics Statement

The study design and sampling methodology was reviewed and approved by the University of Edinburgh Ethics Committee, UK (ERC No: OS02-13) and by the Ministry of Scientific Research and Innovation (MINRESI), Cameroon. MINRESI gave permission to conduct the fieldwork and issued fieldwork permits. The research did not involve endangered of protected species and no further approvals were necessary to conduct fieldwork. All participants gave informed verbal consent to participate and were aware they could opt out at any stage. Verbal consent was deemed appropriate for the variety of dialects spoken, variable literacy amongst participants and due to the remote outdoor fieldwork environment. Information to be provided to participants, for informed verbal consent, was communicated to the interviewer in a written document. Additional training was provided to the interviewer regarding the consent procedure and interview process. Furthermore, the interviewer was experienced in conducting questionnaires in similar studies and spoke the various local dialects of study participants. Verbal consent was recorded on a cover sheet to the questionnaire by the interviewer and refusals were recorded in separate document along with reasons for refusal.

## Author Contributions

BB, VT, KM, and LN designed the original study in Cameroon. RK, SM, and VN collected the samples in the field. J-MB, RK, and CN carried out the diagnostic tests. IH and BB carried out the statistical analysis. BB, CN, and RK prepared the first draft of the paper and all authors commented.

### Conflict of Interest Statement

The authors declare that the research was conducted in the absence of any commercial or financial relationships that could be construed as a potential conflict of interest.
