# Supplementary material for: Comparison of Two Rift Valley Fever Serological Tests in Cameroonian Cattle Populations Using a Bayesian Latent Class Approach
Source: Front Vet Sci. 2019 Aug 14;6:258. doi: 10.3389/fvets.2019.00258 (PMC6702286; doi:10.3389/fvets.2019.00258)

Comparison of two Rift Valley Fever serological tests in Cameroonian cattle populations using a Bayesian latent class approach – Supplementary material

B.M.deC. Bronsvoort[1,2*], J-M. Bagninbom[3], L. Ndip[4], R.F. Kelly[1,2], I.G. Handel[1,2], V.N. Tanya[5,8], K.L. Morgan[6], V. Ngu Ngwa[3], S. Mazeri[1], C. Nfon[7]

[1] The EERA Group, The Roslin Institute and The Royal (Dick) School of Veterinary Studies, University of Edinburgh, Easter Bush, Midlothian, United Kingdom

[2] The Royal (Dick) School of Veterinary Studies, University of Edinburgh, Easter Bush, Midlothian, United Kingdom

[3] School of Veterinary Medicine and Sciences, B.P. 454, University of Ngaoundere, Ngaoundere, Cameroon

[4] Laboratory of Emerging Infectious Diseases, University of Buea, Buea, Cameroon

[5] Programme Office, Cameroon Academy of Sciences, P.O. Box 1457, Yaoundé, Cameroon

[6] Institute of Ageing and Chronic Disease and School of Veterinary Science, University of Liverpool, Leahurst Campus, Neston, Wirral, United Kingdom

[7] National Centre for Foreign Animal Disease, Winnipeg, Manitoba, Canada

[8] Institute of Agricultural Research for Development, Regional Centre of Wakwa, Ngaoundere, Cameroon

*mark.bronsvoort@ed.ac.uk

S1 – Hui-Walter model code with dependence

Example of R code used to run the Hui-Walter conditional dependence model.

```
#reads in the raw data set
rvf <- read.csv(file = "rvf.cambtb20190207.csv", header = TRUE,
stringsAsFactors = FALSE)

#select out variables of interest
rvf <- rvf[ ,c("AnimalID", "HER_ID", "ABREED", "ANIAGE", "ANIDEN" ,
"ANISEX" , "DIVSIO" ,"SUBDIV", "IDVET.RVFAB.CP", "rvf_ELISA_PN40",
"rvf_ELISA_PN50", "strata1",  "strata2", "PRNT_PN80", "PRNT_PN40",
"PRNT80.titre", "ABREED2")]

#create matrix of test result pairs
pop <- t(matrix(as.vector(table(rvf$PRNT_PN80, rvf$rvf_ELISA_PN40,
rvf$strata2)), 4,13))
```

```
# Data table for final test cut-offs selected after initial screening of
different cut-off combinations

      -/-  -/+  +/-  +/+
      [,1] [,2] [,3] [,4]     Sub-Division
 [1,]  140    1    2    7     "BELEL"
 [2,]   80    2    1    0     "BOYO"
 [3,]  179    2    5    8     "BUI"
 [4,]  155    3    6    9     "DONGA MANTUNG"
 [5,]  219    2    3   29     "MARTAP"
 [6,]   26    0    2    2     "MBE"
 [7,]   67    3    1    3     "MENCHUM"
 [8,]   85    5    1    3     "MEZAM"
 [9,]   59    1    0    0     "MOMO"
[10,]   67    1    0    5     "NGAN-HA"
[11,]   60    0    0    0     "NGAOUNDERE"
[12,]   35    1    4    5     “NGOKETUNJIA"
[13,]  165    1    3   11     "NYAMBAKA"


np <- nrow(pop)


#create vector of population counts
n <- apply(pop,1,sum)


#  test order -- / -+ / +- / ++
# test 1 VNT; test 2 ELISA

#the jags model

cat("model{
      for (j in 1:13){
  pop[j, 1:4] ~ dmulti(par[j, 1:4], n[j])
  p[j] ~ dunif(0, 1)
  par[j,4] <- p[j]*(SeRVF_ELISA*SeRVF_PRNT+covDp) + (1-p[j])*((1-
SpRVF_ELISA)*(1-SpRVF_PRNT)+covDn)
  par[j,2] <- p[j]*(SeRVF_ELISA*(1-SeRVF_PRNT)-covDp) + (1-p[j])*((1-
SpRVF_ELISA)*SpRVF_PRNT-covDn)
  par[j,3] <- p[j]*((1-SeRVF_ELISA)*SeRVF_PRNT-covDp) + (1-
p[j])*(SpRVF_ELISA*(1-SpRVF_PRNT)-covDn)
  par[j,1] <- p[j]*((1-SeRVF_ELISA)*(1-SeRVF_PRNT)+covDp) + (1-
p[j])*(SpRVF_ELISA*SpRVF_PRNT+covDn)
  }

  ls <- (SeRVF_ELISA-1)*(1-SeRVF_PRNT)
  us <- min(SeRVF_ELISA,SeRVF_PRNT) - SeRVF_ELISA*SeRVF_PRNT
  lc <- (SpRVF_ELISA-1)*(1-SpRVF_PRNT)
  uc <- min(SpRVF_ELISA,SpRVF_PRNT) - SpRVF_ELISA*SpRVF_PRNT
  rhoD <- covDp / sqrt(SeRVF_ELISA*(1-SeRVF_ELISA)*SeRVF_PRNT*(1-
SeRVF_PRNT))
  rhoDc <- covDn / sqrt(SpRVF_ELISA*(1-SpRVF_ELISA)*SpRVF_PRNT*(1-
SpRVF_PRNT))


  SeRVF_ELISA ~ dbeta(20,1)
  SpRVF_ELISA ~ dbeta(5,1)
  SeRVF_PRNT ~ dbeta(5,2)
```

```
  SpRVF_PRNT ~ dbeta(10,2)
  covDn ~ dunif(lc, uc)
  covDp ~ dunif(ls, us)

}", file="NGS2T.jag")
```

# initial values for the 3 chains

```
INI <- list(
            list(SeRVF_ELISA=0.8, SpRVF_ELISA=0.55, SeRVF_PRNT= 0.6,
SpRVF_PRNT=0.8,p=runif(length(n), 0, 0.35)),
            list(SeRVF_ELISA=0.55, SpRVF_ELISA=0.95, SeRVF_PRNT= 0.67,
SpRVF_PRNT=0.98, p=runif(length(n), 0, 0.35)),
            list(SeRVF_ELISA=0.7, SpRVF_ELISA=0.9, SeRVF_PRNT= 0.7,
SpRVF_PRNT=0.9, p=runif(length(n), 0, 0.35)))
```

#compile all the model components

```
M <- jags.model(data=list(pop=pop,n=n), inits=INI, n.chains=3, n.adapt=
50000, file="NGS2T.jag")
```

#run the model with 50,000 burn in then 250,000 iterations and 100 fold thinning

```
R <- coda.samples(M, c("SeRVF_ELISA", "SpRVF_ELISA","SeRVF_PRNT",
"SpRVF_PRNT", "p", "covDn", "covDp"), n.iter=250000, n.thin=100)
lablist <- c("SeRVF_ELISA", "SeRVF_PRNT", "SpRVF_ELISA", "SpRVF_PRNT",
"covDn", "covDp", "BELEL", "BOYO", "BUI", "DONGA MANTUNG", "MARTAP", "MBE",
"MENCHUM","MEZAM","MOMO","NGAN-HA", "NGAOUNDERE", "NGOKETUNJIA",
"NYAMBAKA")


# Convergence checks
# pop1 best fit

 gelman.diag(R)
 gelman.plot(R)
 densityplot(R)
 traceplot(R)
```

S2 Table – Overall test accuracy for different test cut-off combinations of the ID.vet RVF ELISA and the in-house $PRNT_{80}$

Test cut-off combination	Se	Sp	Accuracy (se+sp-1)
**ID.Vet RVF ELISA**			
PRNT_PN80/rvf_ELISA_PN40	0.856	0.986	0.842
PRNT_PN40/rvf_ELISA_PN40	0.894	0.944	0.838
PRNT_PN80/rvf_ELISA_PN50	0.766	0.985	0.731
PRNT_PN40/rvf_ELISA_PN50	0.802	0.940	0.742
**In house RVF $PRNT_{80}$**			
PRNT_PN80/rvf_ELISA_PN40	0.844	0.981	0.825
PRNT_PN40/rvf_ELISA_PN40	0.608	0.968	0.576
PRNT_PN80/rvf_ELISA_PN50	0.857	0.975	0.832
PRNT_PN40/rvf_ELISA_PN50	0.818	0.975	0.793

The combined accuracy of the two tests for a given set of cut-offs was then compared and using PRNT_PN80/rvf_ELISA_PN40 had the highest overall accuracy at 0.842+0.825-1=0.667.

S3 Sensitivity analysis

Gelman-Rubin plots for the sensitivities and specificies for the final model showing satisfactory convergance.

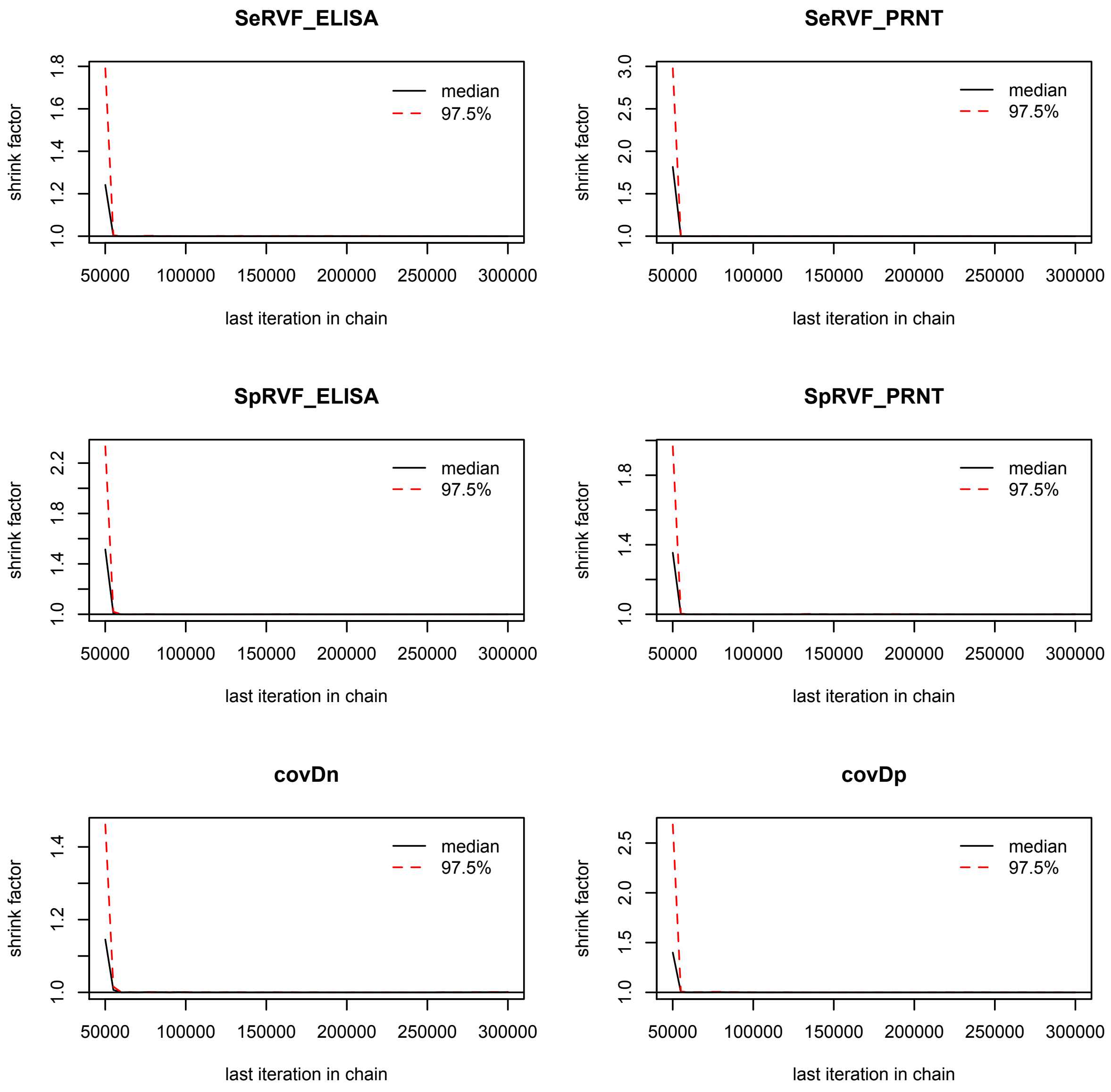

Summary of sensitivity analysis

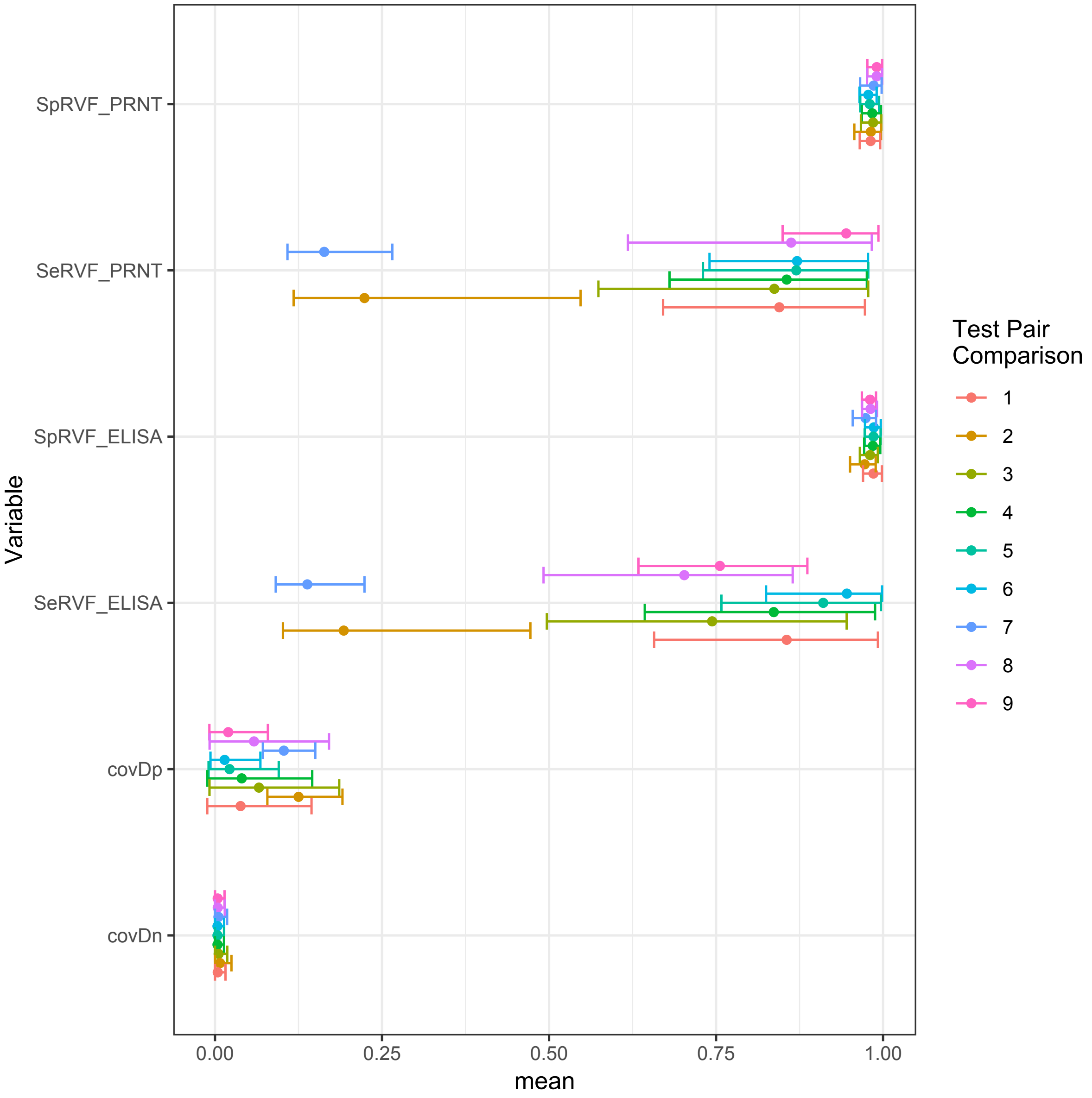

A range of priors were examined to assess the sensitivity of the results (posterior distributions) to these priors. In order to have an identifiable solution with the extra parameters that have to be estimated in the dependence model and to avoid the well recognized problem of bimodal solutions it was not possible to use vague beta(1,1) priors for all parameters. Given that the seroprevalences were unknown and we had no prior information for these we choice to include relatively informative priors for the test parameters of sensitivity and specificity. It was felt that a reasonable assumption was that even though the tests were unlikely to perform in this population as well as in the more controlled test development setting it would be reasonable to believe that they were likely to have sensitivity and specificities >50%.

Test set 1 are the priors used in the final model in the paper and in the figure is can be seen that the data has strong support for much higher and precise specificies and this is seen through so will not be mentioned again. The ELISA Se has a slightly lower and more uncertain estimate that the priors showing that the priors are not overwhelming the data

while the PRNT posterior is more precise and higher than that of the prior again showing there is information in the data and they are not overwhelmed by the priors.

In set 2 the priors were all made vaguer and this results in the sensitivity and prevalences flipping (the prevalences are not shown for space) and given that we really don't believe the test are likely to perform this poorly we believe these are not useful priors.

In set 3 we used more informative priors across the parameters and this produced reasonable posteriors however, the PRNT posterior and the prior appear to completely overlap suggesting the prior may be overly influential in this model.

In set 4 we increased the strength of the priors for the specificities to check that this had no impact on the sensitivity estimates which is it did not have.

In set 5 and 6 we increased the strength of the prior on the ELISA Se to check for any influence on the PRNT posterior estimates which did not seem to be the case. However the ELISA Se posterior is over whelmed by the prior.

In set 7, 8 and 9 we allowed the ELISA Se to be anything between 0 and 1 and explored the impact of different priors for the PRNT Se. At the beta(5,2) we again have a problem of identifiability and the se and prevalence flip. Increase the strength of the priors on the PRNT produce believable estimates for both sensitivities but the posteriors for the PRNT Se are overwhelmed by the priors.

Set 1 priors (final model)
SeRVF_ELISA ~ dbeta(20,1)
SpRVF_ELISA ~ dbeta(5,1)
SeRVF_PRNT ~ dbeta(5,2)
SpRVF_PRNT ~ dbeta(10,2)

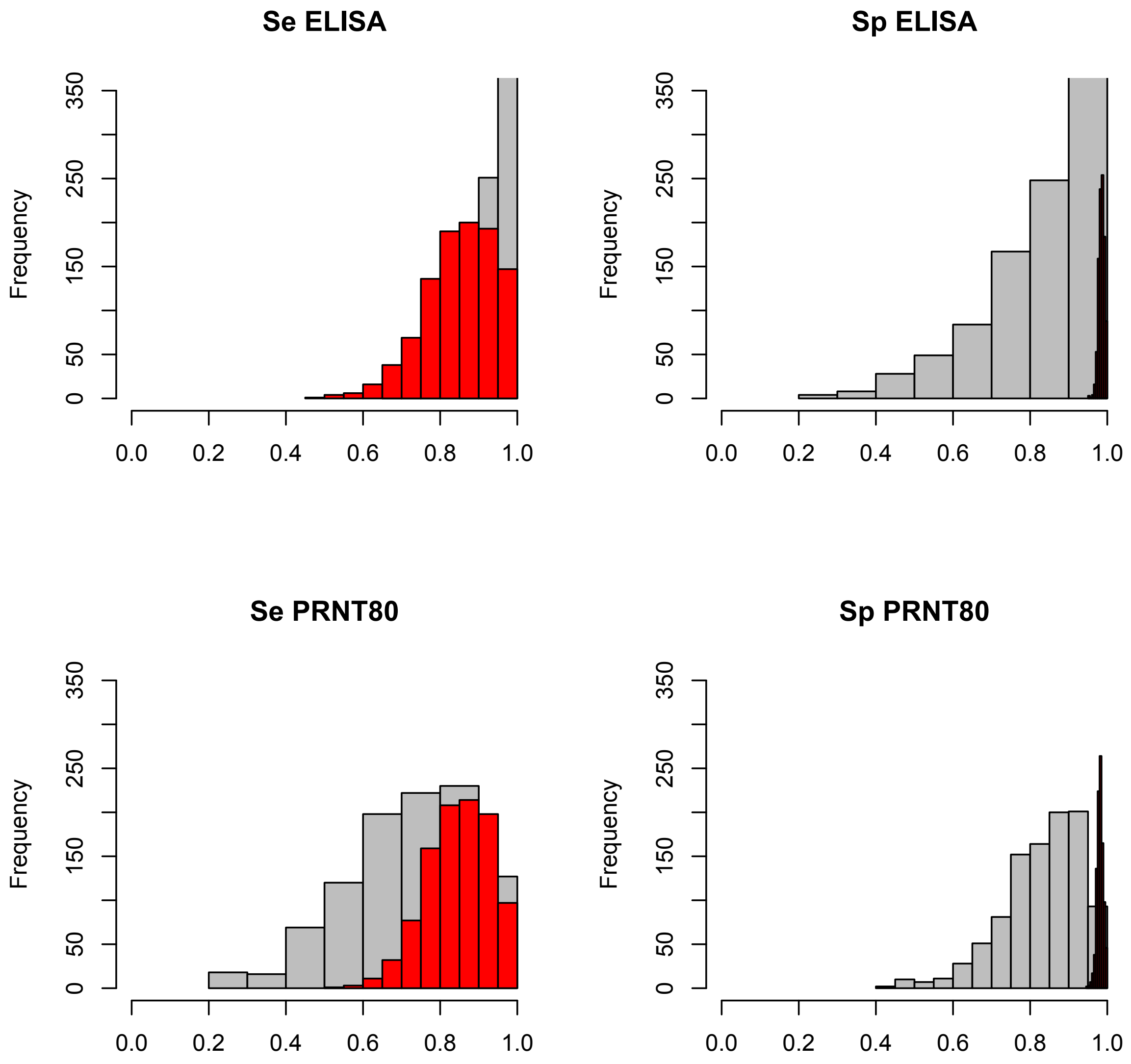

Set 2 priors
SeRVF_ELISA ~ dbeta(5,1)
SpRVF_ELISA ~ dbeta(5,2)
SeRVF_PRNT ~ dbeta(5,2)
SpRVF_PRNT ~ dbeta(5,2)

These priors get too vague and allow the prevalences to switch to >80% and the sensitivity to fall below 50%. Since we have prior believe that the seroprevalence can not be this high and the tests not that bad we would not want to use these priors.

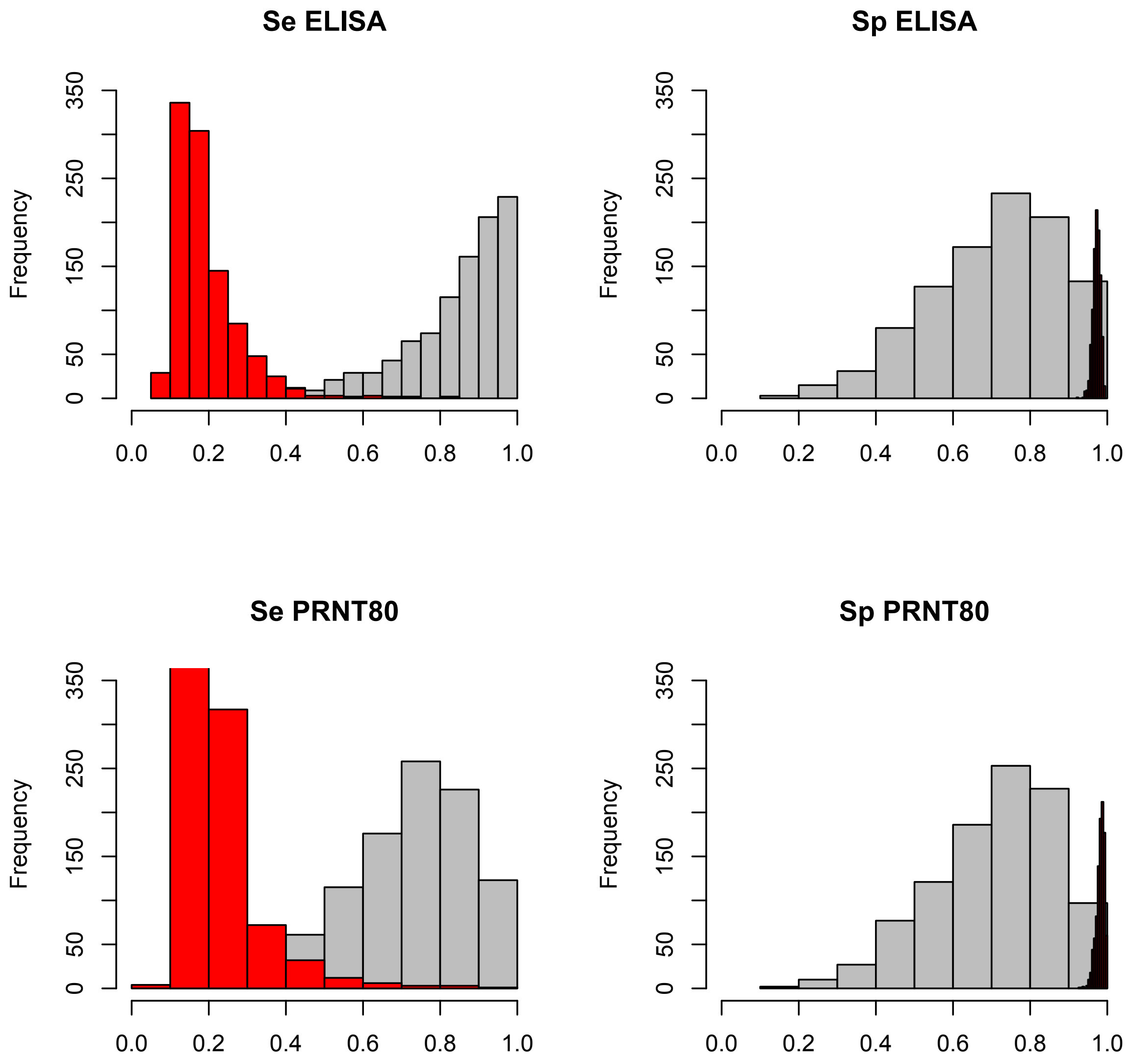

Set 3 priors
SeRVF_ELISA ~ dbeta(10,1)
SpRVF_ELISA ~ dbeta(10,2)
SeRVF_PRNT ~ dbeta(10,2)
SpRVF_PRNT ~ dbeta(10,2)

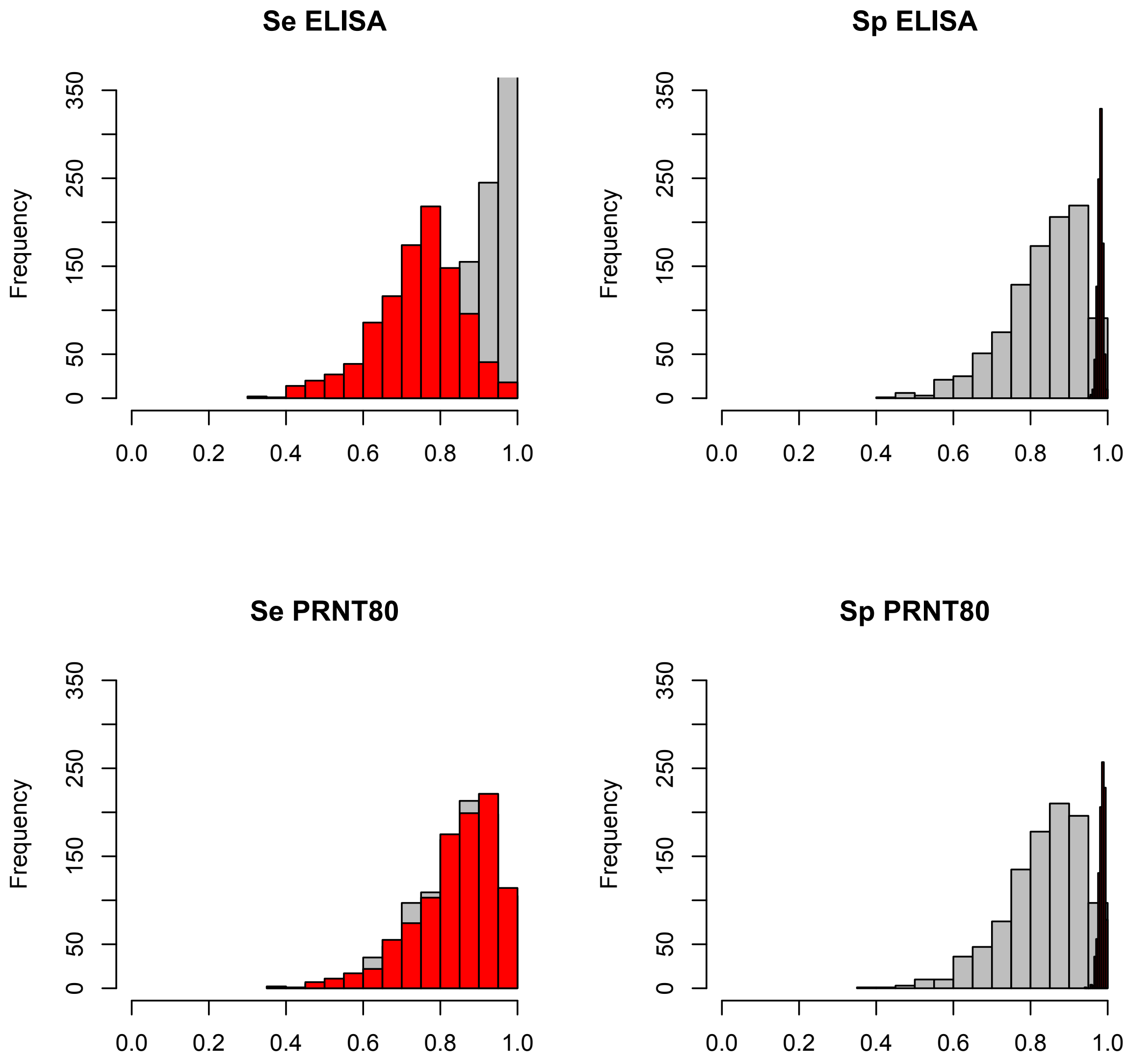

Set 4 priors
SeRVF_ELISA ~ dbeta(20,1)
SpRVF_ELISA ~ dbeta(50,2)
SeRVF_PRNT ~ dbeta(5,2)
SpRVF_PRNT ~ dbeta(50,2)

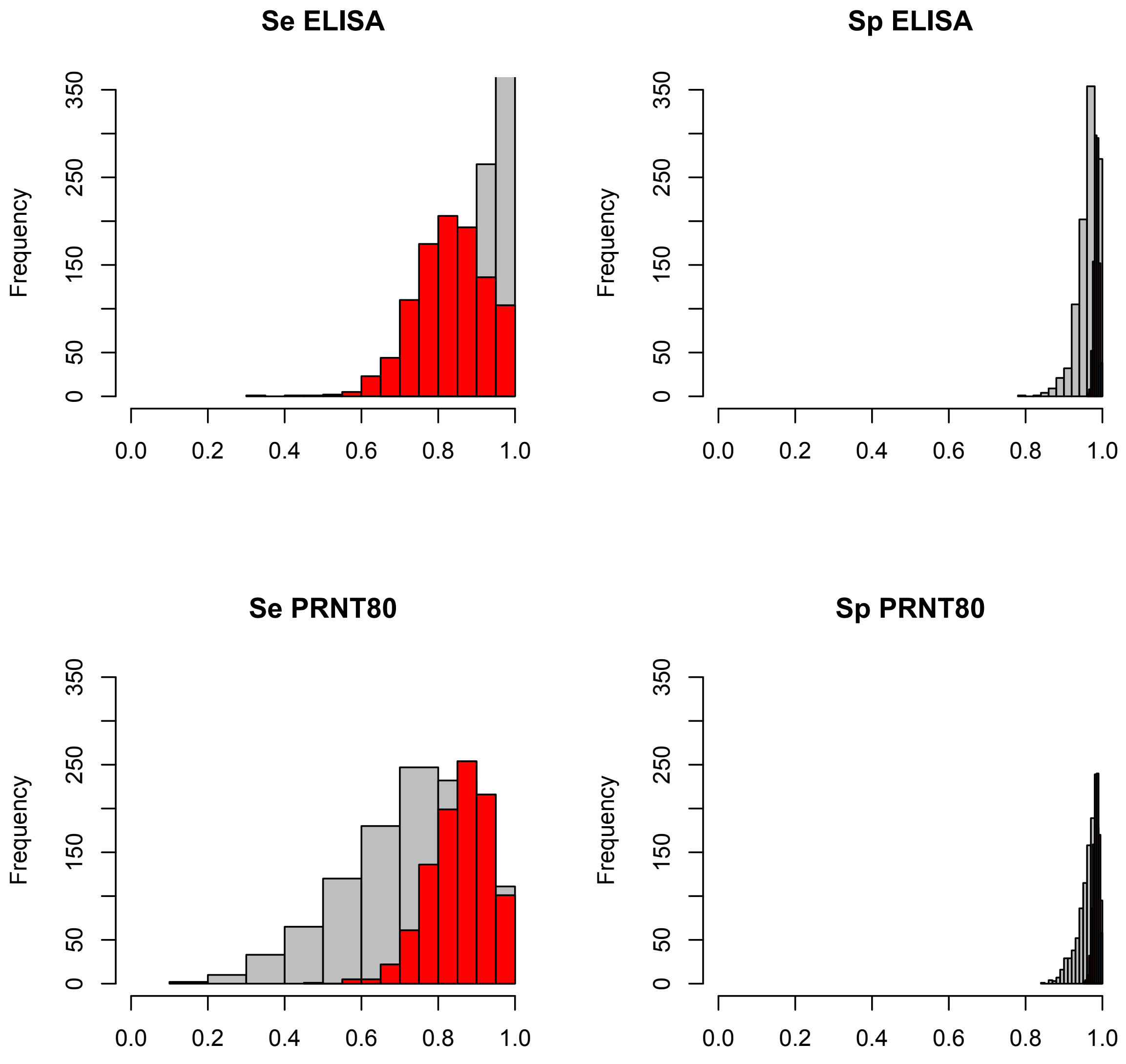

Set 5 priors
SeRVF_ELISA ~ dbeta(30,1)
SpRVF_ELISA ~ dbeta(50,2)
SeRVF_PRNT ~ dbeta(5,2)
SpRVF_PRNT ~ dbeta(50,2)

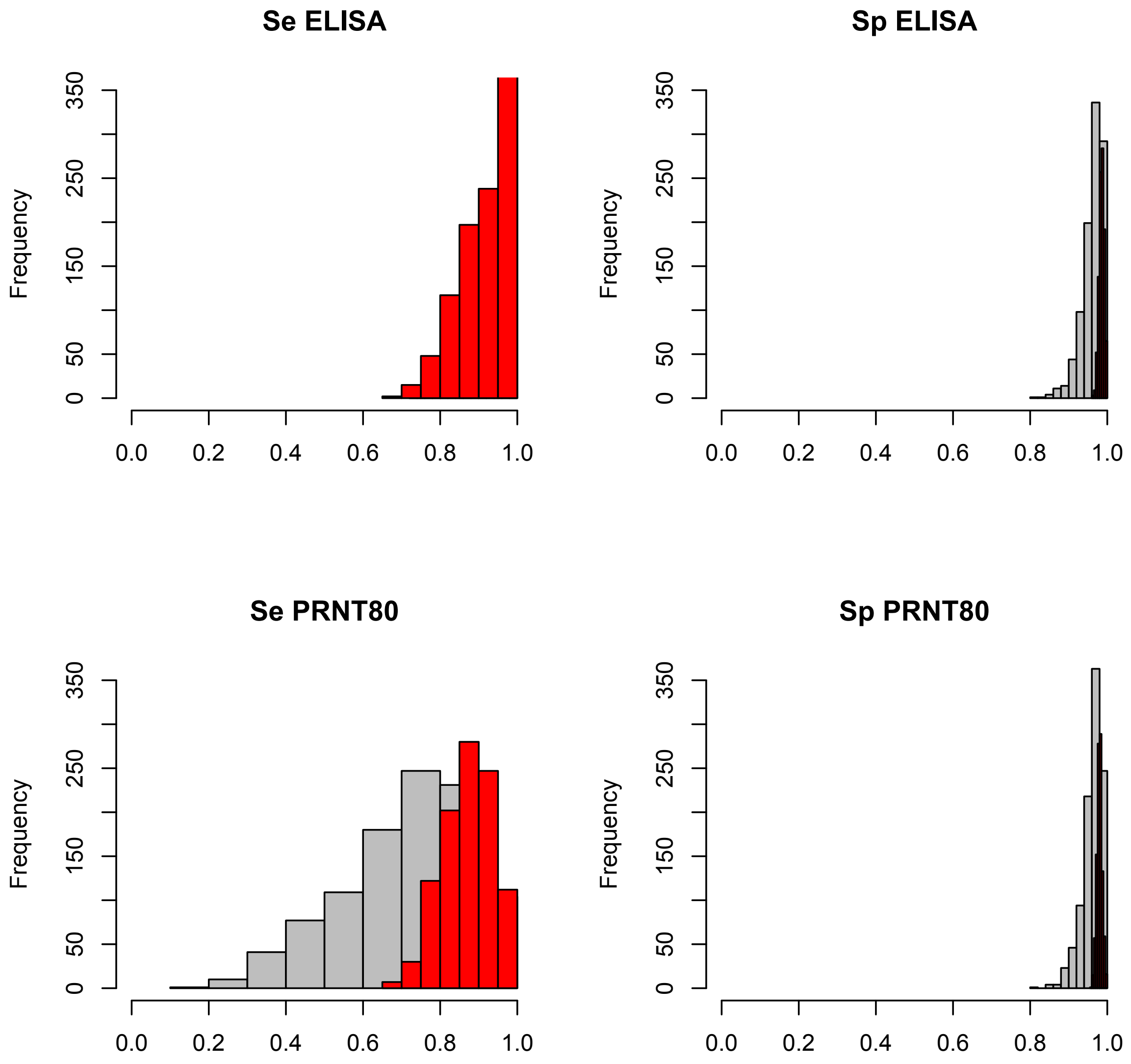

Set 6 priors
SeRVF_ELISA ~ dbeta(40,1)
SpRVF_ELISA ~ dbeta(50,2)
SeRVF_PRNT ~ dbeta(5,2)
SpRVF_PRNT ~ dbeta(50,2)

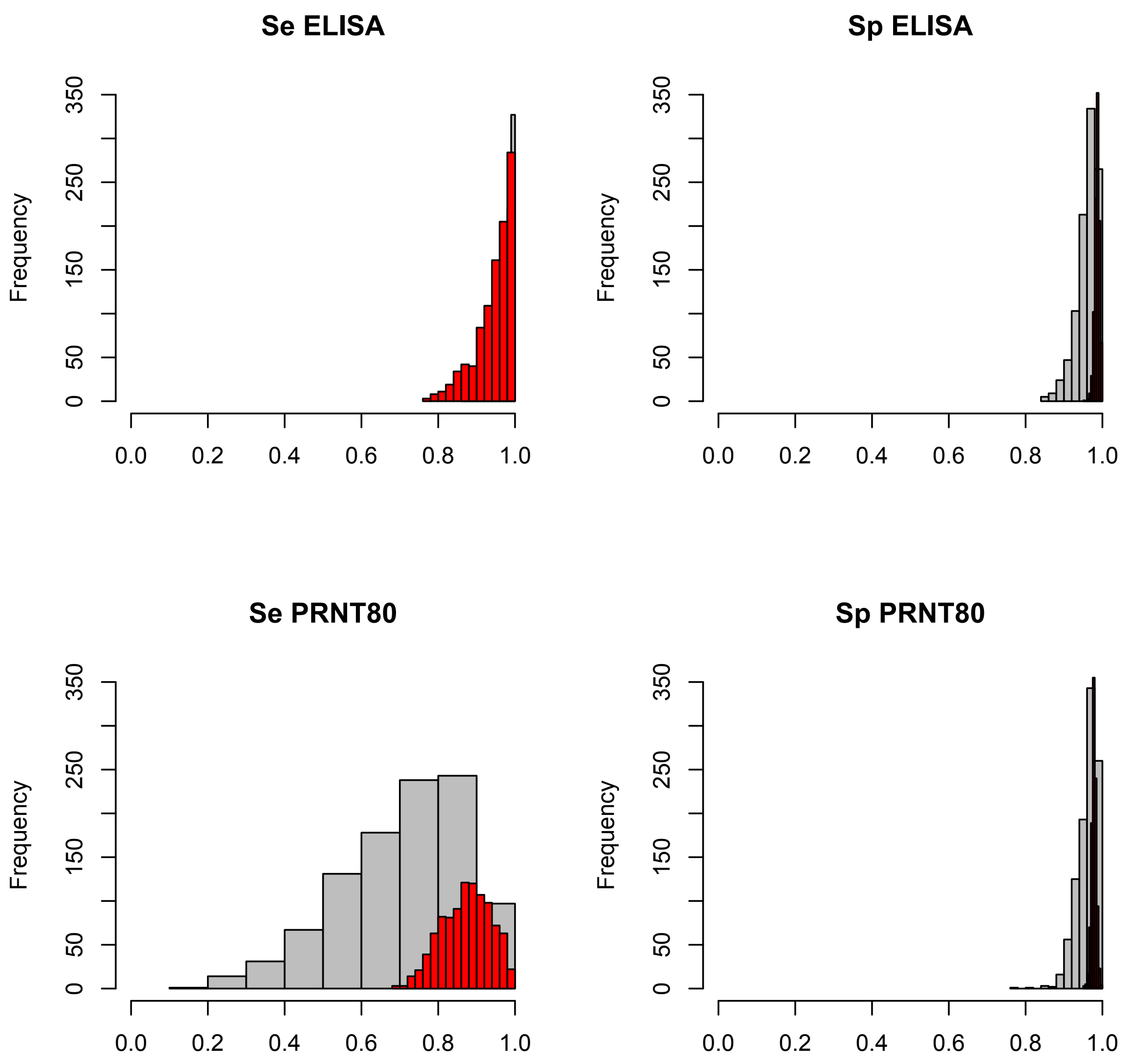

Set 7 priors
SeRVF_ELISA ~ dbeta(1,1)
SpRVF_ELISA ~ dbeta(50,2)
SeRVF_PRNT ~ dbeta(5,2)
SpRVF_PRNT ~ dbeta(50,2)

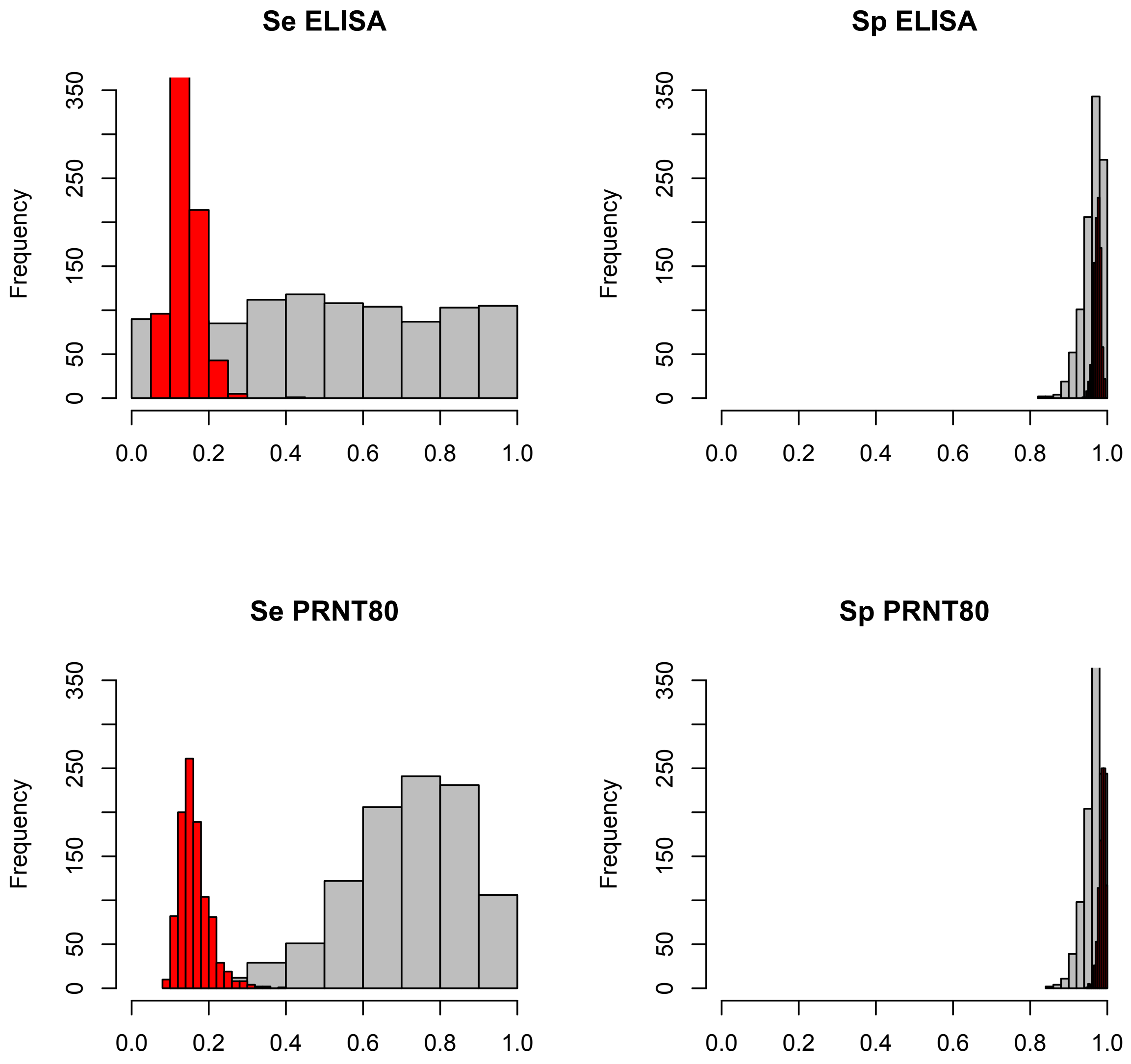

Set 8 priors
SeRVF_ELISA ~ dbeta(1,1)
SpRVF_ELISA ~ dbeta(50,2)
SeRVF_PRNT ~ dbeta(20,2)
SpRVF_PRNT ~ dbeta(50,2)

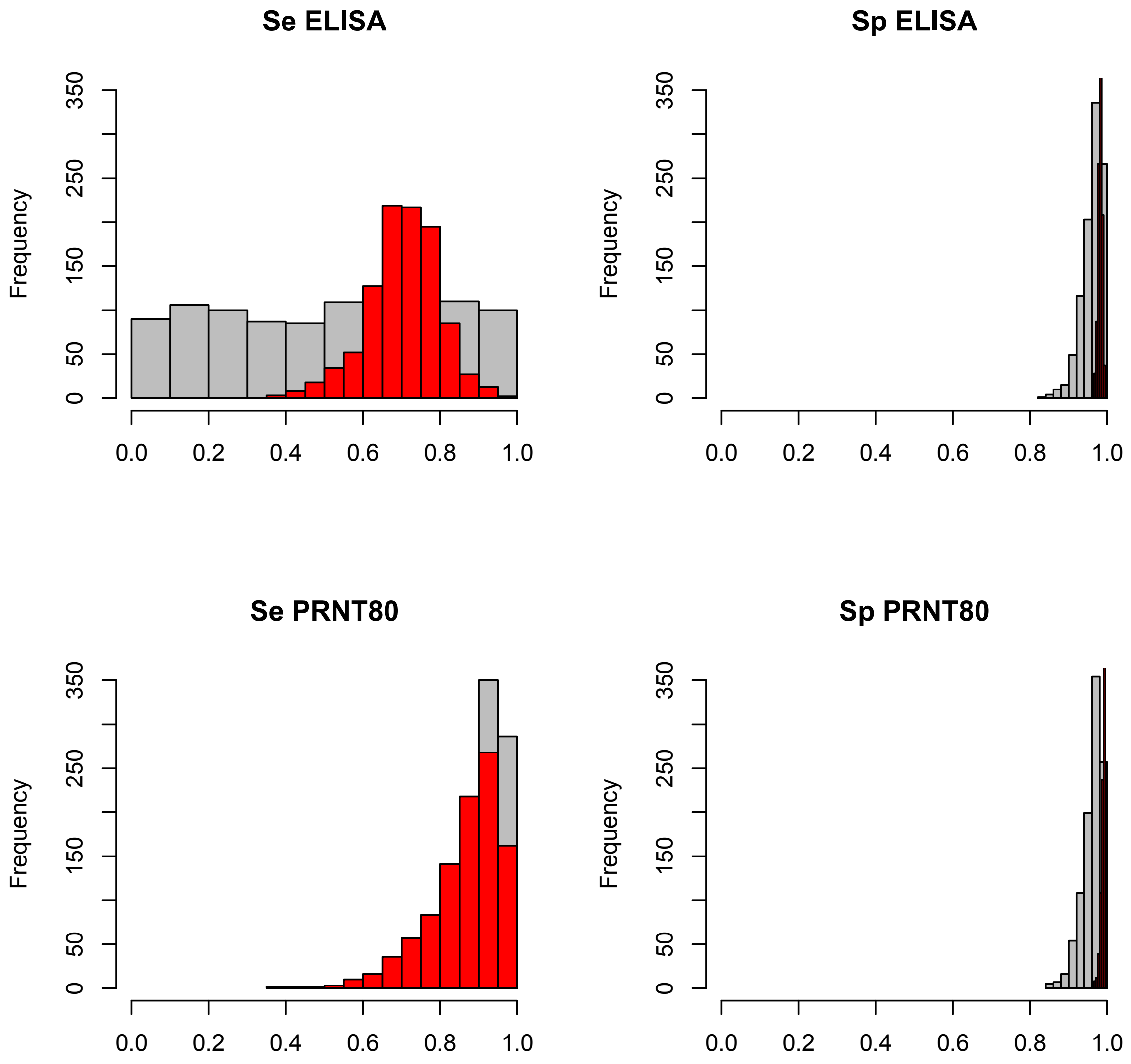

Set 9 priors
SeRVF_ELISA ~ dbeta(1,1)
SpRVF_ELISA ~ dbeta(50,2)
SeRVF_PRNT ~ dbeta(40,2)
SpRVF_PRNT ~ dbeta(50,2)

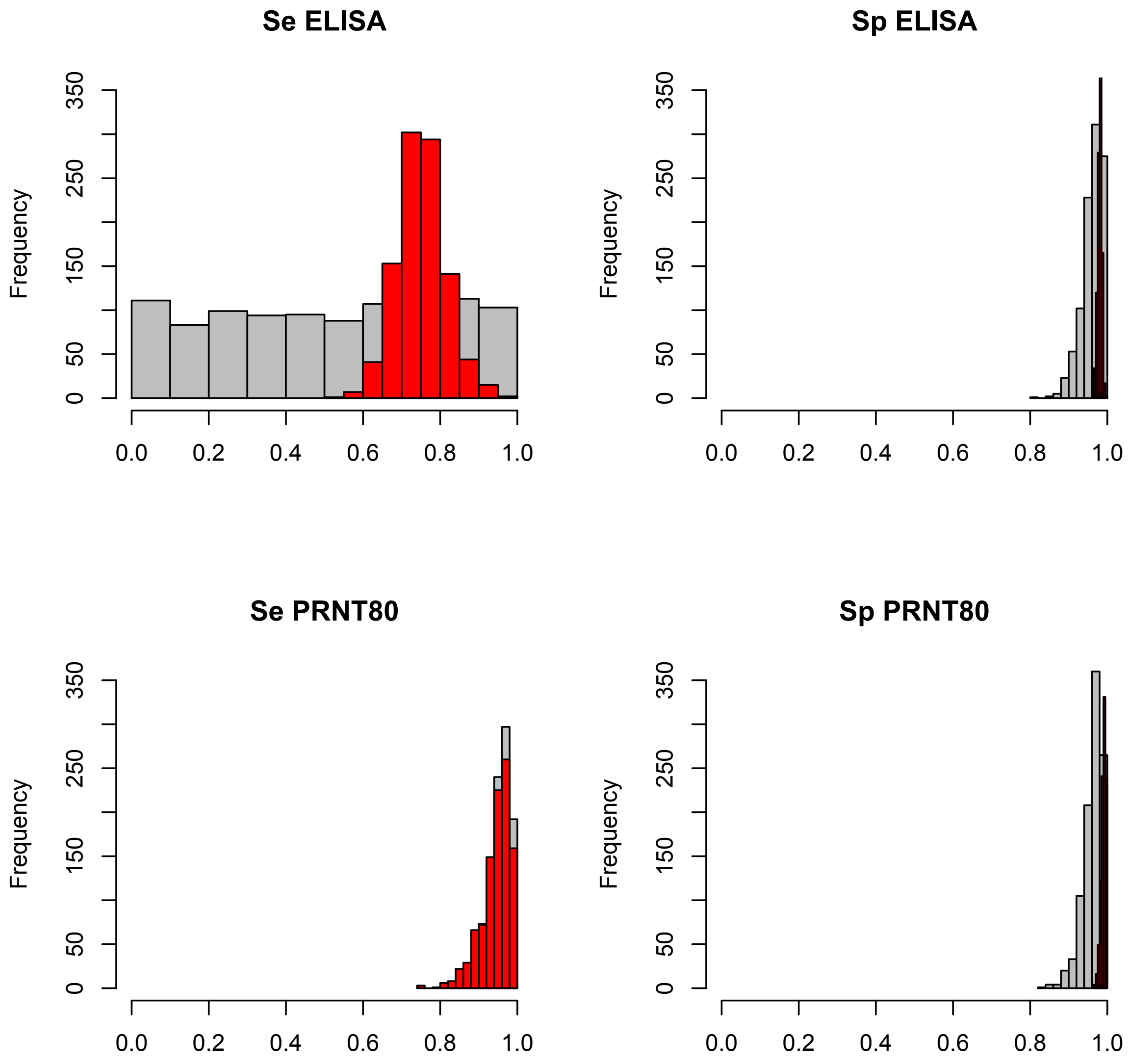

Supplement: Supplementary file 1 [file Presentation_1.pdf]
